# Changes in peripheral T-lymphocyte subsets and serum cytokines in patients with systemic sclerosis

**DOI:** 10.3389/fphar.2022.986199

**Published:** 2022-11-03

**Authors:** Rong-Hong Guo, Hao Cheng, Xiao-Ying Zhang, Zhen Yu, Guang-Hui Wang, Shu-Ya Hao, Xiao-Peng Gao, Hong-Yan Wen

**Affiliations:** Department of Rheumatology, Shanxi Medical University, The Second Hospital of Shanxi Medical University, Taiyuan, Shanxi, China

**Keywords:** T-lymphocyte subsets, cytokines, systemic sclerosis, interstitial lung disease (ILD), arthralgia

## Abstract

**Objective:** T cells represent a predominant cell type in autoimmune disease. However, their exact roles are not fully clear in systemic sclerosis (SSc). This study aimed to mainly investigate the alteration in the absolute numbers of T-lymphocyte subsets and the serum levels of cytokines in SSc patients.

**Methods:** A total of 76 patients with SSc and 76 age- and sex-matched healthy controls (HCs) were enrolled. The levels of circulating T cell subsets and serum cytokines were measured by flow cytometry. T cell subsets or serum cytokines correlations with disease activity and organ involvement were analyzed.

**Results:** The absolute numbers of Th2 and Treg cells in SSc patients were lower than those in HCs (*p* < 0.05), resulting in the ratios of Th1/Th2 [25.01 (12.24, 38.61) vs. 11.64 (6.38, 20.34)] and Th17/Treg [0.42 (0.17, 0.66) vs. 0.17 (0.13, 0.29)] were increased significantly (*p* < 0.001). The absolute numbers of total T, Th, and Treg cells were negatively correlated with CRP (*r* = -0.406, *p* = 0.002; *r* = -0.263, *p* < 0.05; *r* = -0.367 *p* < 0.01). The serum levels of IL-2, SIL-2R, IL-6, IL-10, INF-γ, and TNF-α were significantly higher than those in HCs (*p* < 0.001). Increasing IL-2 in the wake of the augment of ESR (*r* = 0.671, *p* = 0.004), so did IL-6 (*r* = 0.378, *p* < 0.05). The ratio of Th17/Treg in SSc-ILD patients had lower levels than that in other patients [0.35 (0.14, 0.53) vs. 0.64 (0.26, 0.93) *p* = 0.028]; Treg cells were lessened in patients with Raynaud’s phenomenon relative to controls [3.00 (2.41, 4.28) vs. 3.55 (2.86, 4.53) *p* < 0.05]. The levels of IL-2, IL-10 and INF-γ [3.32 (1.05,11.73) vs. 2.32 (0.44,6.45), *p* = 0.045], [8.08 (3.63, 355,77) vs. 4.89 (0.78, 21.44), *p* = 0.02], [6.31 (2.66, 44.03) vs. 4.03 (0.22, 16.96), *p* = 0.009] were elevated in patients with arthralgia, while the level of Th17 was decreased [0.62 (0.20,2.16) vs. 1.26 (0.22,10.93), *p* = 0.026]. ROC curve analysis yielded an optimal cut-off IL-2, IL-10, and INF-γ levels of 2.67, 5.93, and 5.32 pg/ml for the presence of arthralgia.

**Conclusion:** We exhibited abnormalities in T subsets and the production of their cytokines in SSc, as compared with those in HCs. This may allow the pathogenesis of SSc and the development of novel therapeutic interventions aimed at targeting these cells and the cytokines they produce.

## Introduction

Systemic sclerosis (SSc) is a complex and heterogeneous autoimmune connective disease characterized by vascular abnormalities, autoimmunity, and extensive fibrosis of the skin and internal organs. It is universally accepted that the pathophysiology of SSc was a progressive self-amplifying process, which first started the microvascular damage, then run an autoimmune response, and was followed by progressive fibrosis (Cutolo et al., 2019). There is growing evidence of epigenetic abnormalities in this intractable disease, including specific modifications affecting the three main cell types that influence SSc pathogenesis: immune cells, endothelial cells, and fibroblasts (Tsou et al., 2021). Many patients have higher mortality due to vital organ involvement such as interstitial fibrosis, pulmonary hypertension, and renal crisis, which can also symptoms that severely affect the quality of life, such as Raynaud’s phenomenon, digital ulcerations, and arthralgia (Georges et al., 2006). It is a challenge for physicians who diagnose in the early oligosymptomatic stage of SSc and understand the pathogenesis for developing new targeted therapies (Herrick et al., 2017).

T cells have been reported to modulate the development of autoimmunity, inflammation, and fibrosis by the secretion of cytokines. It also has been found to be necessary to produce autoantibodies against a variety of nuclear proteins in SSc (Stochmal et al., 2020). Studies conducted in the progression of SSc have led to the recognition that T helper (TH) cells are involved in the early inflammatory response and late fibrotic phases by producing cytokines and interacting with fibroblasts (O'Reilly et al., 2012). Abnormal levels of T cell-derived cytokines, which act as humoral mediators during the immune response, have been found in the serum of SSc patients (Baraut et al., 2012). The concept of polarization of Th1/Th2 has been widely known as the classical pattern, Th1 cell-derived cytokines (such as IFN-γ, TNF-α, IL-1, IL-2, and IL-12) participate in pro-inflammatory responses, whereas Th2 cell-derived cytokines (IL-4, IL-13, IL-5, IL-6, and IL-10) promote the overproduction of collagen by fibroblasts (Baraut et al., 2010). Thus, Th2 is mostly involved in fibrosis, whereas Th1 rather induces inflammation at the early stages of SSc. Indeed, several cell types can produce cytokines, including CD8^+^ T, dendritic, Th17, and regulator T (Treg) cells. Understanding the mechanisms that generate this pattern of immune response and analyzing the levels of cytokines as diagnostic and prognostic markers in SSc patients is significant.

This study aimed to investigate the alteration of peripheral T-lymphocyte subpopulations and serum cytokines in SSc patients. We exhibited abnormalities in these cells and the production of cytokines by them, as compared with NCs. We observed the correlation between cytokines with disease activity and organ involvement. Furthermore, abnormal expression of cytokines has joint diagnostic significance. This may allow the pathogenesis of SSc and the development of novel therapeutic interventions aimed at targeting these cells and the cytokine they produce.

## Materials and methods

### Patients and controls

A total of 76 SSc patients enrolled, who visited the Rheumatology and Immunology Department of the Second Hospital of Shanxi Medical University from January 2018 to December 2020, and enrolled in the study were initial treatment and aged between 18 and 80 years. The diagnosis of SSc was based on the American College of Rheumatology/European League Against Rheumatism (ACR/EULAR) classification criteria (van den Hoogen et al., 2013). The patients were excluded from this study if they were suffering from malignant disease, had a history of malignancy, had a recent clinically significant infection, or had any other connective tissue disease. We also included 76 healthy controls (HCs), who visited the physical examination center of our hospital during the same period, had no rheumatic immunological and family history, and were matched with SSc patients in age and gender. Each subject provided a signed consent form. Peripheral blood and serum samples were taken; the clinical and laboratory indexes and auto-antibodies were detected using freshly blood samples at the same time. Our study was approved by the Ethics Committee of the Shanxi Medical University Second Affiliated Hospital and all candidates have signed informed consent. (No.2019YX009).

### Clinical measurements

The clinical data of SSc patients were collected through retrospective medical records. Disease duration, which is the clinical period of the disease, was defined as the period from the first appearance of symptoms and signs to this visit. Esophageal dysfunction was defined as symptoms of acid reflux heartburn or findings of reflux esophagitis on gastrointestinal endoscopy. Cardiovascular diseases were assessed by color Doppler echocardiography (EF, ECO, PHILIPS EPIQ7C). The diagnosis of interstitial lung disease (ILD) was determined by a review of a high-resolution computed tomography scan (HRCT, GE DiscoveryRT). Lung involvement and fibrosis were evaluated using HRCT, which has four features: single ground glass lesions, ground glass lesions and reticular nodular lesions (mixed pattern), single reticular nodular lesions (reticular-nodular), and honeycomb changes. Diagnosed renal involvement with blood pressure exceeding 140/90 mmHg and decreased renal function (Renal dysfunction was defined as an increase in serum creatinine concentration of more than 30% above baseline levels at any study time point). In addition, the presence of joint contractures and Raynaud’s phenomenon were noted.

### Flow cytometry

Th1, Th2, and Th17 Cell cultures and Labeling: An 80 μl blood sample together with 10 μl phorbol myristate acetate working solution (final concentration, 30 ng/ml), 10 μl ionomycin working solution (final concentration, 750 ng/ml), and 1 μl GolgiStop was incubated at 37°C and 5% CO2 for 5 h. The samples were then divided into two tubes, followed by staining with anti-CD4-FITC antibodies at room temperature in the dark for 30 min. To the tubes was added 1 ml freshly prepared fixation/permeabilization solution; the tubes were then placed in an incubator at 4°C in the dark for 30 min. Anti-IL-4-PE and anti-interferon gamma (IFN-γ)-APC were added to tube A; Anti-FITC-CD4 and anti-IFN-γ-APC (intracellular staining) were used to detect Th1 cells, while anti-FITC-CD4, and anti-IL-4-PE (intracellular staining) were used to detect Th2 cells. Anti-human IL-17-PE (intracellular staining) was added to tube B for Th17-cell analysis. The two tubes of cells were stored at room temperature for 30 min in the dark and then washed with phosphate-buffered saline (PBS). The absolute numbers of CD4^+^ T lymphocyte subsets were automatically detected using BD Multitest software (BD Biosciences, Franklin Lakes, NJ, United States). All immunofluorescence antibodies were purchased from BD Biosciences.

Detection of Treg Cells: Anti-CD4-FITC and anti-CD25-APC were added to an 80 μl blood sample and incubated at room temperature in the dark for 30 min. Then, 1 ml freshly prepared fixation/permeabilization solution was added to each tube, mixed, and incubated at 4°C for 30 min AntiFOXP3-PE (intracellular staining) was added and incubated at room temperature for 30 min in the dark, followed by washing with PBS and detection of Treg cells using flow cytometry. All immunofluorescence antibodies were purchased from BD Biosciences.

Flow cytometry: The stained cells were measured using flow cytometry (Calibur; BD Biosciences) within 24 h. Based on the scatter plot of the forward angular scattered light relative to the lateral angular dispersive light (side scatter (SSC)), the lymphocytes were gated to distinguish them. CD4 was used to distinguish CD4^+^ T cells from the SSc gate; 10,000 cells from the gate were taken. The relative percentages were obtained and analyzed using CellQuest software. The absolute number of cells in each subgroup was calculated using the following equation: absolute cell number = percentage of positive cells in each subset × the absolute number of CD4^+^ T cells (cells/μl) cells/μl whole blood.

### Measurement of laboratory assessment

C3 and C4 complement levels, total serum immunoglobulin titers, and Erythrocyte Sedim entation Rate (ESR) were measured by nephelometry. The serum was separated from 4 ml venous blood after 1–2 h and stored at −20°C. The levels of serum cytokines [interleukin-2 (IL-2), IL-4, IL-6, IL-10, tumor necrosis factor-α (TNF-α), interferon-γ (IFN-γ), and IL-17] in SSc were measured using flow cytometry. A cytometric bead array (CBA) kit was purchased from Jiangsu Sage Biotechnology Co. Ltd. (Jiangsu, China) and used according to the manufacturer’s instructions; the results were expressed as pg/ml.

### Statistical analysis

Dichotomous variables were expressed as percentages and absolute frequencies, and continuous features were expressed as mean ± standard deviation (SD) or median four quantile method [M (P25, P75)]. The count data were measured using the chi-square goodness-of-fit test. The independent sample *t*-test was used for comparisons between the two groups. Nonnormal distribution data were performed using the Mann–Whitney’s *U*-test for the comparisons of differences between groups, using the Kruskal-Wallis H test among multiple groups. Correlation analysis was performed using Spearman’s rank correlation coefficient. All reported *p* values were 2-sided and were not adjusted for multiple testing. The level of significance was set at *p* < 0.05. All analyses were conducted using SPSS software version 26.0 (IBM, Armonk, NY, United States).

## Results

### Patients’ disposition and baseline characteristics

Clinical data on the 76 patients with SSc were shown in Table 1. Of the 76 patients, 9.21% were male, and 90.79% were female. The average age of disease onset was 56.47 ± 12.42 years. The average hospitalization time was 15.60 ± 10.09 days. The course of the disease was 8.00 (3.00, 11.00) years. SSc was clinically classified into three groups: patients with diffuse cutaneous SSc (dcSSc) (65, 85.83%), an overlapping syndrome in which multiple diseases occur simultaneously (9, 11.84%), and limited cutaneous SSc (lcSSc) (2, 2.63%). Our cohort of patients with SSc represented untreated patients with diverse disease presentations, with some patients having none of the organs affected (32.89%), and others having a more systemic multiorgan disease (e.g., cardiovascular diseases, pulmonary disease, gastrointestinal disease**,** and renal involvement) (see Table 1). The proportion of patients who had ILD was 65.79%, while patients with cardiovascular diseases were 11.84%. Other involvement included arthralgia and Raynaud’s phenomenon were 31.58% and 86.84% in the study subjects respectively. Anti-nuclear antibodies were positive in 76.32% of the patients. Among them, 7.89%, 25%**,** and 10.53% were also positive for anti-centromere antibodies (ACA), anti-Scl-70 antibodies, and anti- Ro52 antibodies, respectively (Table 1). The values of each inflammatory indicator in SSc patients and HCs were shown in Table 1. Among them, ESR (22.50 (13.00,47.00) vs. 9.00 (5.00,14.00) mm/h) and CRP (6.92 (2.01,13.05) vs. 2.89 (1.33,3.56) mg/L) in SSc were higher compared with HCs. However, the value of PLT (198.50 (153.00,265.25) *109/L vs. 238.00 (212.00,291.50) *109/L) was the opposite.

**TABLE 1:** Overview of patients with SSc and healthy controls (HCs).

Clinical data	SSc patients	HCs	*p* Value
Age at disease onset (years)	56.47 ± 12.42	53.63 ± 8.10	0.097
The hospitalization time (days)	15.60 ± 10.09	—	—
The course of the disease (years)	8.00 (3.00,11.00)	—	—
Male	9.21% (7/76)	10.53% (8/76)	0.786
Female	90.79% (69/76)	89.47% (68/76)	0.786
ESR (mm/h)	22.50 (13.00,47.00)	9.00 (5.00,14.00)	**<0.001**
CRP (mg/L)	6.92 (2.01,13.05)	2.89 (1.33,3.56)	**<0.001**
Immunoglobulin G (g/L)	11.80 (9.28,14.15)	10.65 (8.61,12.45)	0.107
PLT (*10^9^/L)	198.50 (153.00,265.25)	238.00 (212.00,291.50)	**0.008**
C3 (g/L)	0.78 (00.67,0.89)	—	—
C4 (g/L)	0.17 (0.13,0.21)	—	—
Anti-nuclear antibodies	76.32% (58/76)	—	—
Anti-centromere antibodies	7.89% (6/76)	—	—
Anti-Scl-70 antibodies	25% (19/76)	—	—
Anti-Ro52 antibodies	10.53% (8/76)	—	—
No. of organs affected	32.89% (26/76)	—	—
Organ involvement	67.1% (51/76)	—	—
Cardiovascular diseases	11.8% (7/76)	—	—
Pulmonary disease	65.78% (50/76)	—	—
Gastrointestinal disease	11.8% (7/76)	—	—
Renal involvement	9.21% (7/76)	—	—
Arthralgia	31.58% (24/76)	—	—
Raynaud’s phenomenon	86.84% (66/76)	—	—
diffuse cutaneous SSc (dcSSc)	85.83% (65/76)	—	—
overlapping syndrome	11.84% (5/76)	—	—
limited cutaneous SSc (lcSSc)	2.63% (2/76)	—	—

*Among those with active disease. Results are given as mean ± SD, M (P25, P75) or percentage; ESR, erythrocyte sedimentation rate; CRP, C-reactive protein; PLT, platelet; Anti-Scl-70, Anti-topoisomerase I antibody; Bold values mean statistical significance.

### Differences in T-lymphocyte subsets and cytokines between patients and healthy controls

**FIGURE 1:**
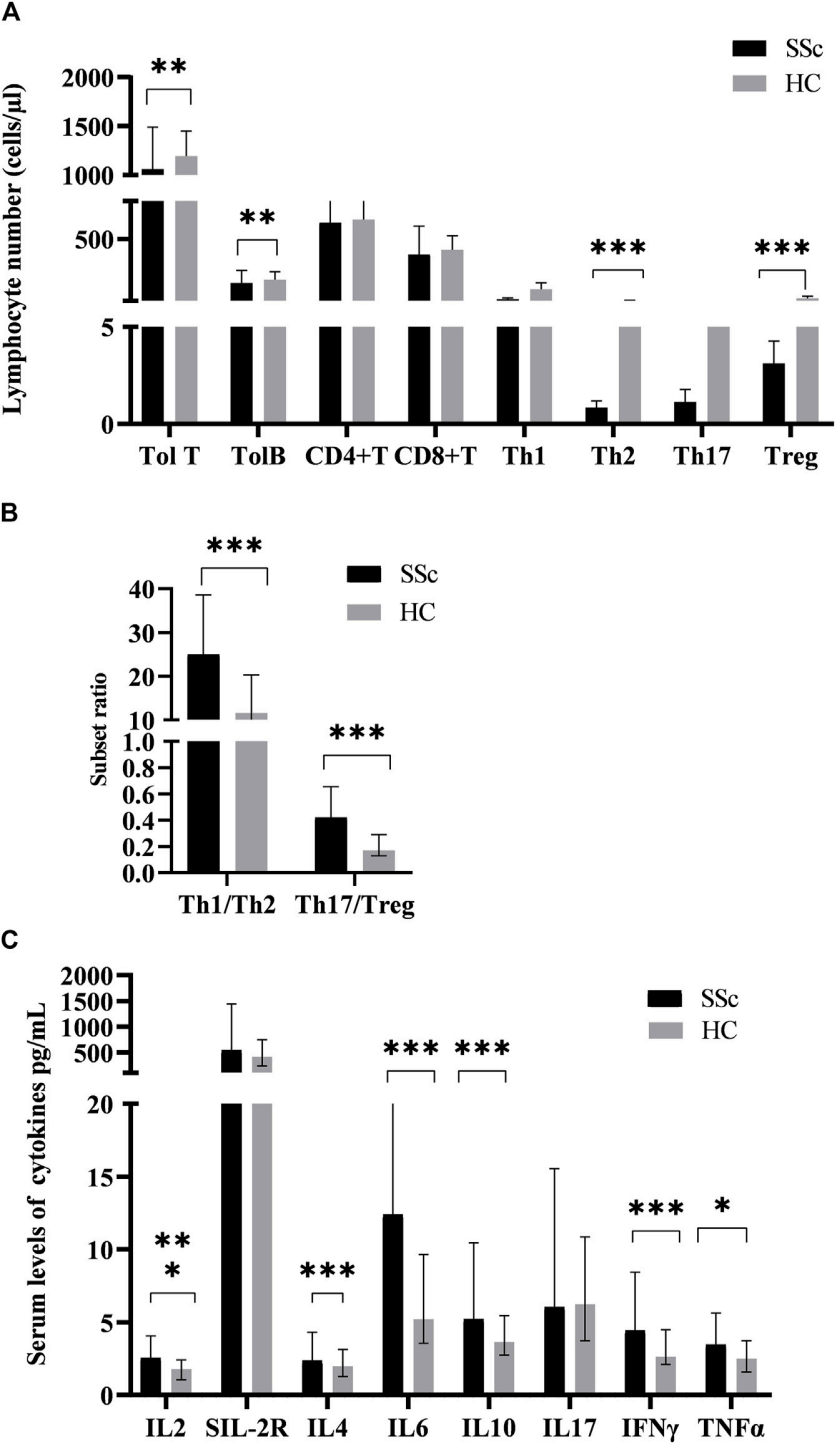
The levels of peripheral lymphocyte subsets and serum cytokines in SSc patients and HCs. **(A)**The absolute numbers of total T (Tol T), total B (Tol B), Th2, and Treg cells in SSc were significantly lower than those in HCs. **(B)**The ratios of Th1/Th2 and Th17/Treg in SSc were significantly higher than those in HCs. **(C)**The serum levels of cytokines in SSc were significantly increased. HC, Healthy controls; SSc, systemic sclerosis; *p < 0.05; **p < 0.01; ***p < 0.001 by Mann–Whitney’s *U*-test.

The values of T-lymphocyte subsets and cytokines in SSc patients and HCs were shown in Figure 1. For analyzing the changes of the adaptive immune cells in peripheral blood in SSc patients, we simultaneously quantified the T and B cells of 76 patients. Although the numbers of both T and B cells were prominent, the absolute numbers of T cells were greater than those of B cells. Among them, total T cells in SSc were lower than those in HCs. Given the varied literature regarding CD4+T cell subsets in the context of SSc, we directly quantified CD4+T cell subsets in patients. We observed that the absolute numbers of Th2 and Treg cells were all decreased in SSc (*p* < 0.001, *p* < 0.001, respectively); their percentages came up with similar results. Interestingly, the absolute numbers of Th1 and Th17 had no significant change. In addition, the ratios of Th1/Th2 and Th17/Treg were comparable between the two groups. Th1/Th2 [25.01 (12.24, 38.61) vs. 11.64 (6.38, 20.34), *p* < 0.001] and Th17/Treg [0.42 (0.17, 0.66) vs. 0.17 (0.13, 0.29), *p* < 0.001] in SSc were higher compared with HCs. These results suggested that T-lymphocyte subsets seem to be involved in the pathogenesis of SSc.

Serum interleukin-2 (IL-2) levels in SSc patients [2.55 (1.05,2.41) pg/ml] were significantly higher than those in HCs [1.78 (1.05,2.41) pg/ml] (*p* < 0.001, Figure 1B). Similarly, serum levels of soluble interleukin-2 receptor (sIL-2R) [544.00 (374.00,1442.75) vs. 414.00 (235.50,682.25) pg/ml], interleukin-6 (IL-6) (12.42(5.43,23.05) vs. 5.16 (3.65,9.94)pg/ml), interleukin-10 (IL-10) [5.23(3.96,10.46) vs. 3.66 (2.74,5.50) pg/ml], INF-γ [4.44 (3.15,8.44) vs. 2.65 (2.12,4.56) pg/ml], and TNF-α [3.46 (2.02,5.64) vs. 2.49 (1.60,3.84) pg/ml]had a similar effect. Furthermore, there was no significant difference in serum interleukin-4 (IL-4) and interleukin-17 (IL-17) levels between SSc and HCs (*p* = 0.146; *p* = 0.958). Similarly, it has the significance that such high expression of cytokines in the pathogenesis of SSc cannot be underestimated.

### The correlation of T-lymphocyte subsets and cytokines with inflammatory indexes

**FIGURE 2:**
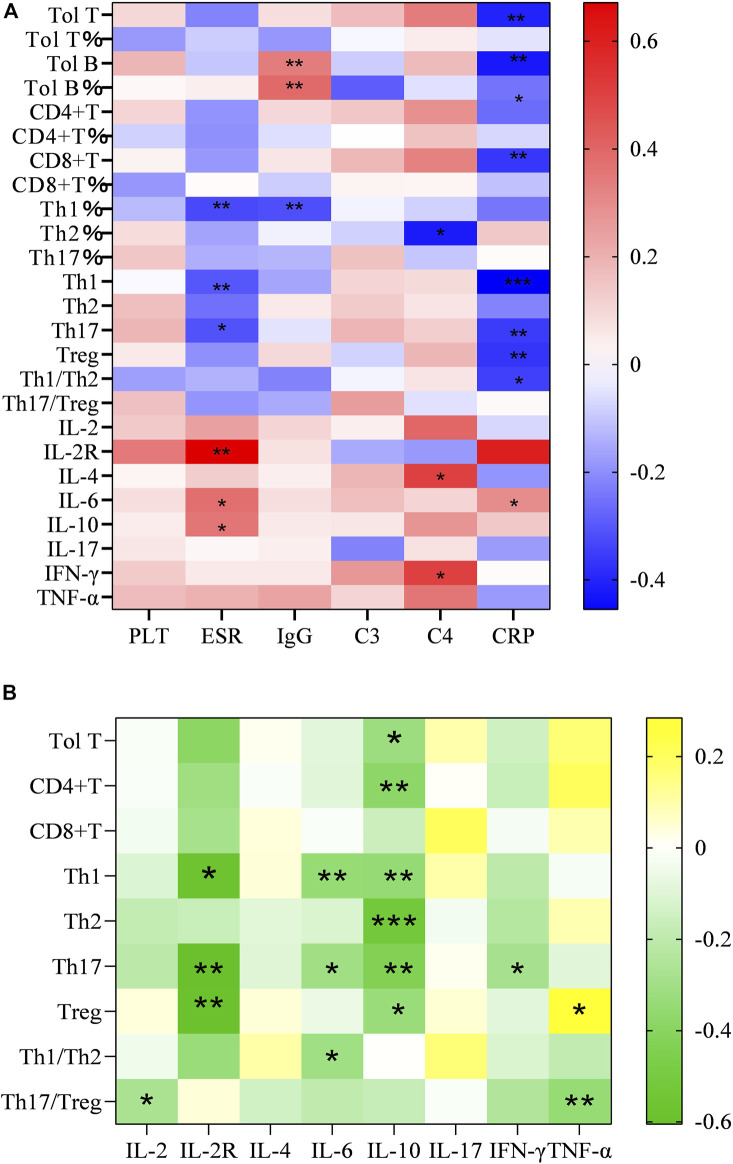
**(A)** Heatmap of correlation of the number and proportion of the circulating T-lymphocyte subsets and serum levels of cytokine with PLT, ESR, CRP, IgG, C3 and C4 in SSc patients; **(B)** Heatmap of correlation of the serum cytokine levels of IL-2, IL-4, IL-6, IL-10, INF-γ, TNF-α with Total T, Th, Th1, Th2, Th17, Treg, Th1/Th2, and Th17/Treg (∗*p* < 0.05, ∗∗*p* < 0.01, and∗∗∗*p* < 0.001 by Spearman’s correlation test).

The serum levels of CRP, ESR, C3, C4, IgG, and PLT tended to be deemed as an indicator of disease activity frequently. As mentioned before, they were higher in the SSc than in the normal group. In addition, we examined the correlation between inflammatory index levels and these T-lymphocyte subsets and cytokines in SSc patients. As a result, total T cells, CD4+T cell, Treg cells and the ratio of Th1/Th2 were negatively correlated with CRP (*r* = −0.406, *p* = 0.002; *r* = −0.263, *p* < 0.05; *r* = −0.367 *p* < 0.01; *r* = −0.346, *p* < 0.05). We found a significantly negative correlation of Th1 cell levels with ESR and CRP (*r* = −0.302, *p* = 0.026; r = −0.455, *p* < 0.001), it equally applied to the relevance of Th17 with ESR and CRP (r = −0.311, *p* = 0.022; r = −0.56, *p* < 0.01). We also analyzed the correlation between other T-cell subsets and inflammatory indicators, but the difference was not statistically significant (Figure 2A).

Analogously, the results emerged a positive correlation between considerable cytokines and inflammatory markers. Increasing sIL-2R in the wake of the augment of ESR (*r* = 0.671, *p* = 0.004), so did IL-6 (*r* = 0.378, *p* < 0.05).

### The correlations of T-lymphocyte subsets and cytokine levels with organ involvements

**FIGURE 3:**
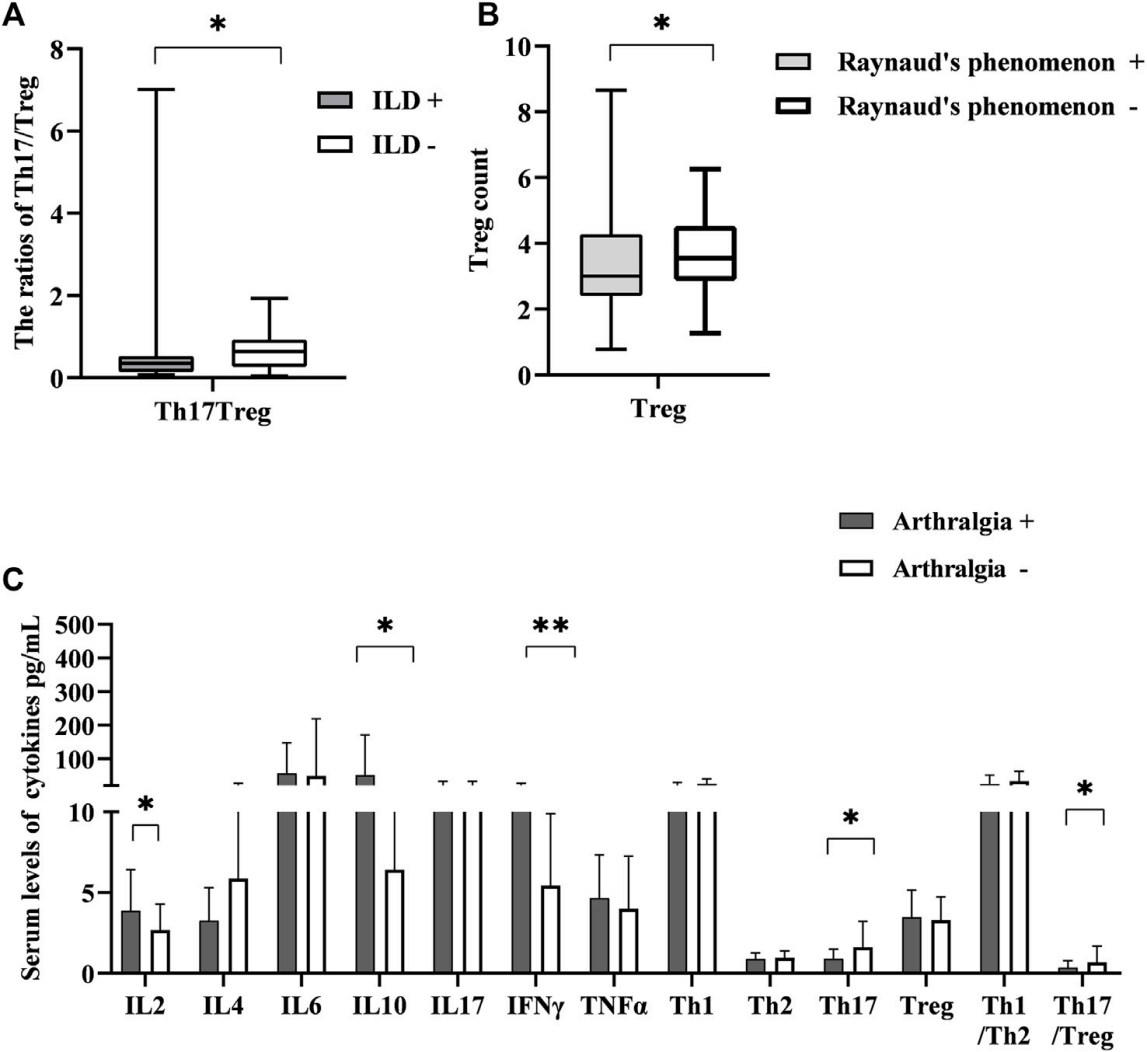
The correlation between the levels of T-lymphocyte subsets and cytokines with different symptoms. **(A)** The ratio of Th17/Treg in SSc patients with interstitial lung disease (ILD) had lower levels than that in those without ILD. **(B)** Treg cell number was lessened in patients with Raynaud’s phenomenon relative to controls. **(C)** The correlation between T-lymphocyte subsets and cytokines levels with the presence of arthralgia. HC, Healthy controls; SSc, systemic sclerosis; *p < 0.05 **p < 0.01, ***p < 0.001 by Mann–Whitney’s *U*-test.

Next, we examined the association between them and organ involvements. For organ involvements, we evaluated the presence of skin sclerosis, interstitial lung disease (ILD), cardiac involvement, esophageal motility disorders, joint contractures, and Raynaud’s phenomenon. In terms of the ILD, only the ratio of Th17/Treg was correlated, and simultaneously had lower levels relative to other patients [0.35 (0.14, 0.53) vs. 0.64 (0.26, 0.93) *p* = 0.028] (Figure 3A). On the other hand, Treg cells were lessened in patients with Raynaud’s phenomenon relative to controls [3.00 (2.41, 4.28) vs. 3.55 (2.86, 4.53) *p* < 0.05] (Figure 3B). We also examined the circulating T-lymphocyte subsets and serum cytokine levels in the presence of arthralgia. IL-2, IL-10 and INF γ [3.32 (1.05, 11.73) vs. 2.32 (0.44, 6.45), *p* = 0.045], [8.08 (3.63, 355,77) vs. 4.89 (0.78, 21.44), *p* = 0.02], [6.31 (2.66, 44.03) vs. 4.03 (0.22, 16.96), *p* = 0.009] were elevated in patients with arthralgia while Th17 was decreased [0.62 (0.20, 2.16) vs. 1.26 (0.22, 10.93), *p* = 0.026]. The ratio of Th17/Treg also decreased [0.21 (0.05, 2.05) vs. 0.45 (0.07, 7.01); *p* = 0.009] (Figure 3C). There were changes in other ratios, but the differences were not statistically significant (not shown, in supplementary data). These findings suggested that the T-lymphocyte subsets and cytokine levels could be potentially useful biomarkers for organ involvement, particularly in arthralgia, which may be an important organ involvement for the early presentation of SSc.

### Serum T-lymphocyte subsets and cytokines for arthralgia

**FIGURE 4:**
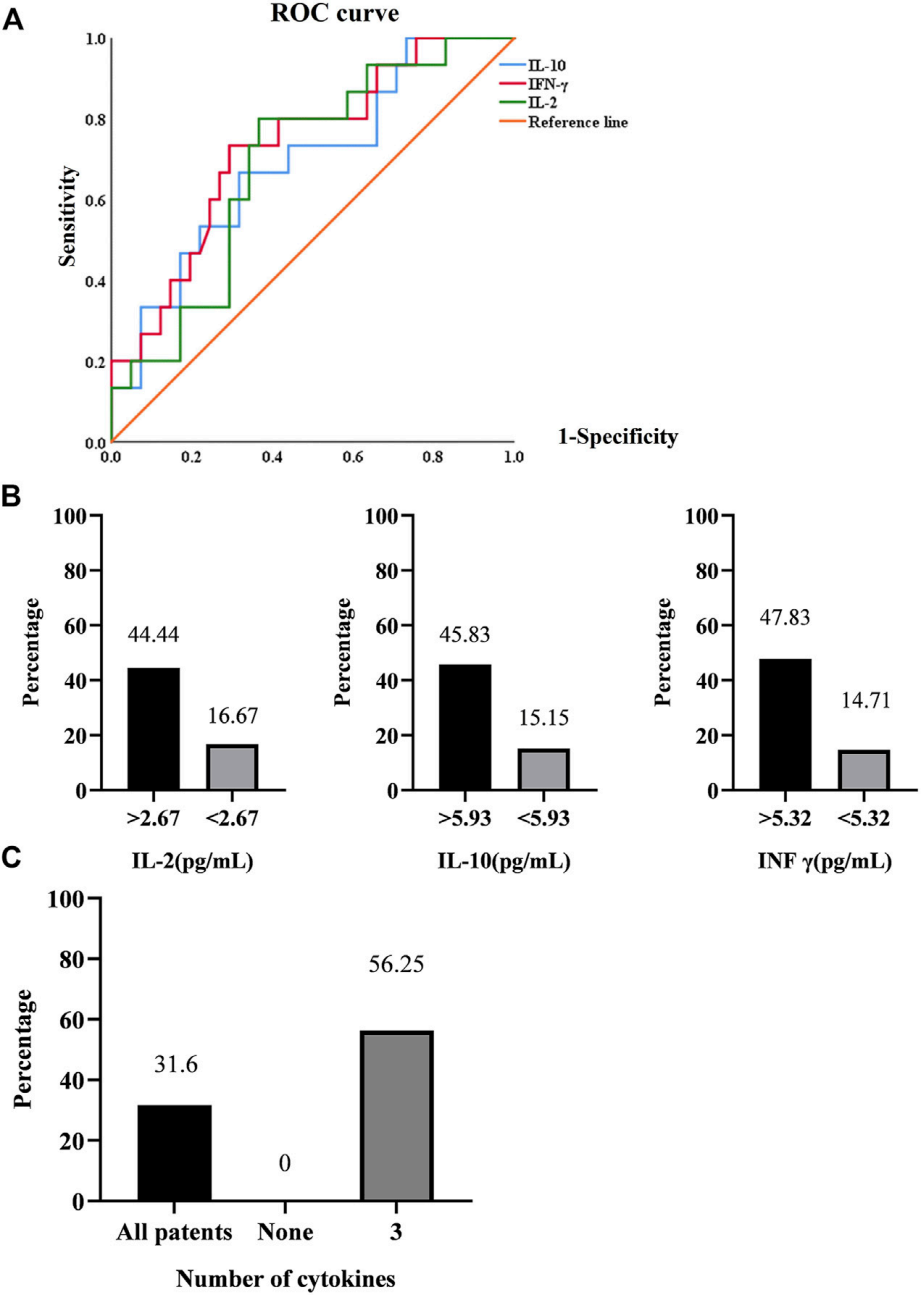
**(A)**. ROC curve analysis in the presence of arthralgia. **(B)**. Prediction rates of arthralgia by IL-2, IL-10, INF-γ. **(C)**. Prediction rates of arthralgia by the synergistic effect of cytokines.

Therefore, we calculated the cut-off value of each cytokine for the presence of arthralgia using ROC curve analysis (Figure 4A). Patients with arthralgia had higher levels of IL-2, IL-10, and INF-γ (Figure 3C). ROC curve analysis yielded an optimal cut-off value (2.67 pg/ml) of IL-2 for the presence of arthralgia. Similarly, levels of IL-10 and INFγ were elevated in patients with arthralgia. (Figure 3C). Subsequent ROC curve analysis yielded an optimal cut-off value of 5.93 and 5.32 pg/ml, respectively (Figure 4A). We then evaluated the usefulness of these optimal thresholds, and as shown in Figure 4B, they all showed a high prevalence of these optimal thresholds. We found that the proportion was significantly higher in patients with IL-2 >2.67 pg/ml; similarly, IL-10, INF-γ. When optimal thresholds for all three cytokines were used, arthralgia was found to have a higher incidence (Figure 4C).

## Discussion

The roles of the immune effector network in the pathogenesis of SSc remain incompletely understood. Our results shed new insights into immune pathologies in SSc by demonstrating the T-lymphocyte subset and cytokine profiles in peripheral blood. First and foremost, we showed the association between dysregulated serum cytokines and altered T-lymphocyte subsets in SSc patients. Furthermore, assessing their correlation with disease activity and organ involvement, we found that abnormal expression of cytokines in joint involvement has joint diagnostic significance. This may allow the development of novel therapeutic strategies aimed at targeting these cells and the cytokine they produce.

It has long been studied that T lymphocytes isolated from the blood or fibrotic skin of SSc patients show oligoclonal libraries (Sakkas et al., 2002). From the perspective of what is known as immunity, the different phenotypes of T cell subsets (such as Th1, Th2, Th17, and Treg) stem from naïve T cells under the action of a variety of cytokine environments. The mediators, released by Th1 and Th2 lymphocytes, can inhibit each other. Th17 and Treg differentiations are interconnected analogously. Indeed, IL-6 plays an important role in determining whether the immune response will be dominated by pro-inflammatory Th17 or by anti-inflammatory Treg cells. The interaction between Th1/Th2 and Th17/Treg also generates a loop effect due to cytokines. IL-17 produced by Th17 can suppress Th1 differentiation (Zhang and Zhang, 2020).

The numbers of CD4^+^ and CD8^+^ T cells represent the predominant T cell subsets in the blood and hardened skin biopsies of SSc patients during the inflammatory phase, as well as the production of pro-fibrotic cytokines such as IL-4 and IL-13 (Fuschiotti, 2016), which play significant roles in activating angiogenesis and promoting collagen production and fibrosis. It has been proposed that an imbalance between the Th1 and Th2 cytokines leads to abnormal responses to tissue damage. Th2 cytokines stimulate the synthesis of collagen, while Th1 cytokines, such as IFN-γ, by contrast, do the opposite (Wynn, 2004). Therefore, the skin or lung fibrosis of SSc can be infiltrated by Th2 rather than Th1 generally (Boin et al., 2008).

However, the reports about Th17 and Treg infiltration in the tissues of SSc patients are controversial. The levels of Th17 cells and cytokines in peripheral blood, skin lesions, and lung tissues of SSc patients have also been found to increase to varying degrees (Rodríguez-Reyna et al., 2012; Zhou et al., 2015), while another study demonstrated their elevated levels of them and a positive correlation with disease activity and collagen overproduction (Yang et al., 2014). Studies have shown that there may be a positive feedback loop betweenTh17 cells that produce IL-17 in SSc, which directly or indirectly stimulates the activation of fibroblasts, vascular endothelial cells, and smooth muscle cells. After activation, cytokines such as IL-6 and IL-8 can enhance the activation of Th17 cells (Liu et al., 2016). The number of Treg cells in peripheral blood can be increased, decreased, or have no difference with weakened function (Boin et al., 2008; Zhou et al., 2015). They participated in autoimmune and tissue fibrosis by producing IL-4 and IL-13.

Different from these, we found that the absolute numbers of Th2 and Treg in blood were lower than those in healthy controls. The distinct results may be affected by factors such as disease course and activity. Considering the limited number of patients in this study, our results need, however, to be validated on a larger number of patients, probably in a multicenter study. Kataoka et al. (2015) found that the decrease of Treg cell subsets and immunosuppressive ability in skin tissues with SSc lesions may be caused by microRNA, DNA methylation, and histone modification affecting FoxP3 (Wang et al., 2014). Notably, patients had higher ratios (imbalance) of Th1/Th2 and Th17/Treg. This result shed light on that T lymphocyte subsets do not wield a single influence independently, rather than play an immune role under the interaction of related circuits or patterns. In addition, our study indicated that there is a general trend of a reduction in T cells and an increase in markers of disease activity (Figure 2A).

Several lines of evidence suggested that effector T cells dominate the inflammatory infiltrates in the involved tissues during the early stages of the disease. Abnormal levels of T cell-derived cytokines, including TNFα, IL-6, IL-10 (Sato et al., 2001), IL-17, IL-4, and IL-13 (Fuschiotti et al., 2009; Hügle et al., 2013; Kang et al., 2019), have been found in the serum of SSc patients. Among these cytokines are thought to promote fibroblast overproduction of collagen, leading to excessive fibrosis. Consistent with previous reports, our conclusion illustrated that most cytokines increased significantly, IL-6 and IL-10 aggrandized particularly is especially obvious. IL-6 is a multifunctional acute-phase inflammatory cytokine that regulates cellular proliferation, activation, and immune responses. It is involved in a wide variety of pathophysiologic processes, such as increasing collagen production and driving differentiation of naive CD4-positive T cells into Th17 cells, which produce inflammatory cytokines (Abdel-Magied et al., 2016). Moreover, it is up-regulation was found to correlate with skin involvement and SSc-ILD (Abdel-Magied et al., 2016)^.^ However, no significant changes in IL-6 in patients with ILD were found in our study. In spite of this, the overwhelming majority of those cytokines showed a positive relationship with activity indexes as shown in Figure 2A.

In order to further investigate whether there are differences in T cells and cytokines among different organ-affected groups, we found that Th17/Treg was lower in patients with ILD, which is quite different from what we previously thought possible. Whereas, Treg has been lower in patients with Raynaud’s phenomenon. Of note, recent studies demonstrated that the progression of the disease can attribute to Treg transformation to pathogenic effector T cells, such as Th17-like or Th2-like cells, which produce inflammatory and profibrotic cytokines respectively (MacDonald et al., 2015; Slobodin and Rimar, 2017). Hence, it is hard not to speculate that there is a transformation of Treg in patients with ILD and Raynaud’s phenomenon. Despite these results, the transformation in the ILD and Raynaud’s phenomenon needs to be understood and clarified.

As mentioned above, the serological IL-2, IL-6 and INF γ levels in the presence of arthralgia elevated, while Th17 and Th17/Treg decreased. We used ROC curves to determine their optimal bounds and, in turn, the optimal bounds were used to predict a higher prevalence. Little is known about IL-10 participation in the pathogenesis of SSc. Almost all leukocytes produce IL-10, which has a short half-life and short range of activity (Saxena et al., 2015). Treg suppresses inflammation and autoimmunity by utilizing IL-10. Furthermore, IL-10 has been shown to downregulate the mRNA expression of type I collagen and fibronectin (Antiga et al., 2010). Similarly, IL-10 was associated with many T lymphocytes in this study (Figure 2B). We also showed high levels of IL-10 and low levels of Treg in patients with arthralgia. The reason for this phenomenon is not only Treg transformation but also IL-10 playing a joint damage process, which of course needs further study.

A cross-sectional analysis of the EUSTAR registry disclosed that the prevalence of articular symptoms was 28% in SSc (Avouac et al., 2010), which frequency is obviously high. Articular symptoms of any form were also found to have a higher incidence of 31.6% in our study. In addition, arthralgia may often occur in the early stages of the disease and was associated with disease activity and inflammatory responses. Jérôme Avouac et al. (2016) conducted a prospective cohort study using the systematic longitudinal follow-up of SSc patients with the EUSTAR registry, which has determined the value of joint synovitis as a predictor of disease progression. Current management of articular involvement is largely supportive and symptomatic. In most cases, simple NSAIDs are effective. Recently, biologics have been widely used to treat inflammatory rheumatism, like using TNF-α inhibitors to get a significant decrease in signs of inflammation or joint symptoms (Lam et al., 2007). However, it is regrettable that randomized controlled trials of SSc were lacking. An expert consensus on the use of TNF-α inhibitors in SSc among EUSTAR centers, which subjected arthritis might respond and should be conducted in more detailed investigation (Distler et al., 2011). Thus, accurate identification of high-risk patients with arthralgia may control the disease progression and optimal efficacy through implementing sufficient treatment at the right time.

A known problem is that it is difficult to distinguish whether joint involvement is an SSc-disease-associated manifestation, an overlap, or accompanying manifestations of an unrelated condition. Polyarticular and patterns of joint involvement have been described (Sandler et al., 2020). This study requires further in-depth research on the classification of joint patterns and the timing of onset.

There are certain limitations to our study. This research sample size is smaller, which recruited a limited number of patients with SSc., and still needs a multicenter, large sample, the long-term clinical observation confirmed that this conclusion. From a clinical point of view, different courses of disease, disease stages, and disease subsets need to be identified to facilitate personalized management approaches. The retrospective study design can also be considered a disadvantage, with treatment guiding decisions rather than preventing physicians from standardizing the purpose of the study background. Therefore, high-quality, large-scale with longer follow-ups are needed to further compare safety and efficacy, and disease stages and other relevant cytokine assays may be increased. However, patient cases represent real-life scenarios, we exhibited abnormalities in T subsets and the production of their cytokines, as compared with those in HCs. These new insights into the pathogenesis of SSc may allow the development of novel therapeutic interventions targeting these cells and the cytokines they produce. Although there is no single direct correlation between cytokine and clinical parameters, they also contribute to the evaluation of SSc patients. These easily detected clinical indicators may be useful for the risk stratification of patients with SSc, therapeutic guidance, and monitoring of disease progression. Moreover, the imbalance or re-balance of T lymphocyte subsets and cytokines may be used as the parameters of diagnosis and therapeutic efficacy.

## Data Availability

The raw data supporting the conclusion of this article will be made available by the authors, without undue reservation.

## References

[B1] Abdel-Magied R. A. Kamel S. R. Said A. F. Ali H. M. Abdel Gawad E. A. Moussa M. M. (2016). Serum interleukin-6 in systemic sclerosis and its correlation with disease parameters and cardiopulmonary involvement. Sarcoidosis Vasc. Diffuse Lung Dis. 33, 321–330. 28079844

[B2] Antiga E. Quaglino P. Bellandi S. Volpi W. Del Bianco E. ComessAtti A. (2010). Regulatory T cells in the skin lesions and blood of patients with systemic sclerosis and morphoea. Br. J. Dermatol. 162, 1056–1063. 10.1111/j.1365-2133.2010.09633.x 20105169

[B3] Avouac J. Walker U. A. Hachulla E. Riemekasten G. Cuomo G. Carreira P. E. (2016). Joint and tendon involvement predict disease progression in systemic sclerosis: A EUSTAR prospective study. Ann. Rheum. Dis. 75, 103–109. 10.1136/annrheumdis-2014-205295 25165035

[B4] Avouac J. Walker U. Tyndall A. Kahan A. Matucci-Cerinic M. Allanore Y. (2010). Characteristics of joint involvement and relationships with systemic inflammation in systemic sclerosis: Results from the EULAR scleroderma trial and research group (EUSTAR) database. J. Rheumatol. 37, 1488–1501. 10.3899/jrheum.091165 20551097

[B5] Baraut J. Farge D. Jean-Louis F. Kesmandt H. Durant C. VerrecchiaF. (2012). Cytokines in systemic sclerosis. Pathol. Biol. 60, 127–139. 10.1016/j.patbio.2009.11.003 20116938

[B6] Baraut J. Michel L. Verrecchia F. Farge D. (2010). Relationship between cytokine profiles and clinical outcomes in patients with systemic sclerosis. Autoimmun. Rev. 10, 65–73. 10.1016/j.autrev.2010.08.003 20713187

[B7] Boin F. De Fanis U. Bartlett S. J. Wigley F. M. Rosen A. Casolaro V. (2008). T cell polarization identifies distinct clinical phenotypes in scleroderma lung disease. Arthritis Rheum. 58, 1165–1174. 10.1002/art.23406 18383361 PMC2662772

[B8] Cutolo M. Soldano S. Smith V. (2019). Pathophysiology of systemic sclerosis: Current understanding and new insights. Expert Rev. Clin. Immunol. 15, 753–764. 10.1080/1744666x.2019.1614915 31046487

[B9] Distler J. H. Jordan S. Airo P. Alegre-Sancho J. J. Allanore Y. Balbir Gurman A. (2011). Is there a role for TNFα antagonists in the treatment of SSc? EUSTAR expert consensus development using the delphi technique. Clin. Exp. Rheumatol. 29, S40–S45. 21586217

[B10] Fuschiotti P. (2016). Current perspectives on the immunopathogenesis of systemic sclerosis. Immunotargets Ther. 5, 21–35. 10.2147/itt.S82037 27529059 PMC4970639

[B11] Fuschiotti P. Medsger T. A. Jr. Morel P. A. (2009). Effector CD8+ T cells in systemic sclerosis patients produce abnormally high levels of interleukin-13 associated with increased skin fibrosis. Arthritis Rheum. 60, 1119–1128. 10.1002/art.24432 19333920

[B12] Georges C. Chassany O. Toledano C. Mouthon L. Tiev K. Meyer O. (2006). Impact of pain in health related quality of life of patients with systemic sclerosis. Rheumatol. Oxf. 45, 1298–1302. 10.1093/rheumatology/kel189 16754629

[B13] Herrick A. L. Pan X. Peytrignet S. Lunt M. Hesselstrand R. Mouthon L. (2017). Treatment outcome in early diffuse cutaneous systemic sclerosis: The European scleroderma observational study (ESOS). Ann. Rheum. Dis. 76, 1207–1218. 10.1136/annrheumdis-2016-210503 28188239 PMC5530354

[B14] Hügle T. O'Reilly S. Simpson R. Kraaij M. D. Bigley V. Collin M. (2013). Tumor necrosis factor-costimulated T lymphocytes from patients with systemic sclerosis trigger collagen production in fibroblasts. Arthritis Rheum. 65, 481–491. 10.1002/art.37738 23045159 PMC6588536

[B15] Kang S. Tanaka T. Narazaki M. Kishimoto T. (2019). Targeting interleukin-6 signaling in clinic. Immunity 50, 1007–1023. 10.1016/j.immuni.2019.03.026 30995492

[B16] Kataoka H. Yasuda S. Fukaya S. Oku K. Horita T. Atsumi T. (2015). Decreased expression of Runx1 and lowered proportion of Foxp3⁺ CD25⁺ CD4⁺ regulatory T cells in systemic sclerosis. Mod. Rheumatol. 25, 90–95. 10.3109/14397595.2014.899736 24716598

[B17] Lam G. K. Hummers L. K. Woods A. Wigley F. M. (2007). Efficacy and safety of etanercept in the treatment of scleroderma-associated joint disease. J. Rheumatol. 34, 1636–1637. 17611970

[B18] Liu M. Wu W. Sun X. Yang J. Xu J. Fu W. (2016). New insights into CD4(+) T cell abnormalities in systemic sclerosis. Cytokine Growth Factor Rev. 28, 31–36. 10.1016/j.cytogfr.2015.12.002 26724976

[B19] MacDonald K. G. Dawson N. A. J. Huang Q. Dunne J. V. Levings M. K. Broady R. (2015). Regulatory T cells produce profibrotic cytokines in the skin of patients with systemic sclerosis. J. Allergy Clin. Immunol. 135, 946-.e9–955. 10.1016/j.jaci.2014.12.1932 25678090

[B20] O'Reilly S. Hügle T. van Laar J. M. (2012). T cells in systemic sclerosis: A reappraisal. Rheumatol. Oxf. 51, 1540–1549. 10.1093/rheumatology/kes090 22577083

[B21] Rodríguez-Reyna T. S. Furuzawa-Carballeda J. Cabiedes J. Fajardo-Hermosillo L. D. Martinez-Reyes C. Diaz-Zamudio M. (2012). Th17 peripheral cells are increased in diffuse cutaneous systemic sclerosis compared with limited illness: A cross-sectional study. Rheumatol. Int. 32, 2653–2660. 10.1007/s00296-011-2056-y 21789610

[B22] Sakkas L. I. Xu B. Artlett C. M. Lu S. Jimenez S. A. Platsoucas C. D. (2002). Oligoclonal T cell expansion in the skin of patients with systemic sclerosis. J. Immunol. 168, 3649–3659. 10.4049/jimmunol.168.7.3649 11907131

[B23] Sandler R. D. Matucci-Cerinic M. Hughes M. (2020). Musculoskeletal hand involvement in systemic sclerosis. Semin. Arthritis Rheum. 50, 329–334. 10.1016/j.semarthrit.2019.11.003 31812353

[B24] Sato S. Hasegawa M. Takehara K. (2001). Serum levels of interleukin-6 and interleukin-10 correlate with total skin thickness score in patients with systemic sclerosis. J. Dermatol. Sci. 27, 140–146. 10.1016/s0923-1811(01)00128-1 11532378

[B25] Saxena A. Khosraviani S. Noel S. Mohan D. Donner T. Hamad A. R. A. (2015). Interleukin-10 paradox: A potent immunoregulatory cytokine that has been difficult to harness for immunotherapy. Cytokine 74, 27–34. 10.1016/j.cyto.2014.10.031 25481648 PMC4454631

[B26] Slobodin G. Rimar D. (2017). Regulatory T cells in systemic sclerosis: A comprehensive review. Clin. Rev. Allergy Immunol. 52, 194–201. 10.1007/s12016-016-8563-6 27318947

[B27] Stochmal A. Czuwara J. Trojanowska M. Rudnicka L. (2020). Antinuclear antibodies in systemic sclerosis: An update. Clin. Rev. Allergy Immunol. 58, 40–51. 10.1007/s12016-018-8718-8 30607749

[B28] Tsou P. S. Varga J. O'Reilly S. (2021). Advances in epigenetics in systemic sclerosis: Molecular mechanisms and therapeutic potential. Nat. Rev. Rheumatol. 17, 596–607. 10.1038/s41584-021-00683-2 34480165

[B29] van den Hoogen F. Khanna D. Fransen J. Johnson S. R. Baron M. Tyndall A. (2013). 2013 classification criteria for systemic sclerosis: An American college of rheumatology/European league against rheumatism collaborative initiative. Ann. Rheum. Dis. 72, 1747–1755. 10.1136/annrheumdis-2013-204424 24092682

[B30] Wang Y. Y. Wang Q. Sun X. H. Liu R. Z. Shu Y. Kanekura T. (2014). DNA hypermethylation of the forkhead box protein 3 (FOXP3) promoter in CD4+ T cells of patients with systemic sclerosis. Br. J. Dermatol. 171, 39–47. 10.1111/bjd.12913 24641670

[B31] Wynn T. A. (2004). Fibrotic disease and the T(H)1/T(H)2 paradigm. Nat. Rev. Immunol. 4, 583–594. 10.1038/nri1412 15286725 PMC2702150

[B32] Yang X. Yang J. Xing X. Wan L. Li M. (2014). Increased frequency of Th17 cells in systemic sclerosis is related to disease activity and collagen overproduction. Arthritis Res. Ther. 16, R4. 10.1186/ar4430 24398084 PMC3979142

[B33] Zhang M. Zhang S. T. (2020). T cells in fibrosis and fibrotic diseases. Front. Immunol. 11, 1142. 10.3389/fimmu.2020.01142 32676074 PMC7333347

[B34] Zhou Y. Hou W. Xu K. Han D. Jiang C. Mou K. (2015). The elevated expression of Th17-related cytokines and receptors is associated with skin lesion severity in early systemic sclerosis. Hum. Immunol. 76, 22–29. 10.1016/j.humimm.2014.12.008 25500255

